# PHAP1 promotes glioma cell proliferation by regulating the Akt/p27/stathmin pathway

**DOI:** 10.1111/jcmm.13639

**Published:** 2018-04-18

**Authors:** Manyi Xie, Zhe Ji, Yaxing Bao, Yufu Zhu, Yang Xu, Lei Wang, Shangfeng Gao, Zhiyi Liu, Zilu Tian, Qingming Meng, Hengliang Shi, Rutong Yu

**Affiliations:** ^1^ Insititute of Nervous System Diseases Xuzhou Medical University Xuzhou China; ^2^ Brain Hospital Affiliated Hospital of Xuzhou Medical University Xuzhou China; ^3^ The Graduate School Xuzhou Medical University Xuzhou China; ^4^ Department of Orthopeadic Surgery First People's Hospital Xuzhou Jiangsu China; ^5^ Department of General Surgery Affiliated Hospital of Xuzhou Medical University Xuzhou Jiangsu China

**Keywords:** Akt, glioma, p27, PHAP1, stathmin

## Abstract

PHAP1 (Putative HLA‐DR‐associated protein 1), also termed acidic leucine‐rich nuclear phosphoprotein 32A (ANP32A), Phosphoprotein 32 (pp32) or protein phosphatase 2A inhibitor (I1PP2A), is a multifunctional protein aberrantly expressed in multiple types of human cancers. However, its expression pattern and clinical relevance in human glioma remain unknown. In this study, Western blotting and immunohistochemistry analysis demonstrated PHAP1 protein was highly expressed in glioma patients, especially in those with high‐grade disease. Publicly available data also revealed high levels of PHAP1 were associated with poor prognosis in glioma patients. The functional studies showed that knock‐down of PHAP1 suppressed the proliferation of glioma cells, while overexpression of PHAP1 facilitated it. The iTRAQ proteomic analysis suggested that stathmin might be a potential downstream target of PHAP1. Consistently, PHAP1 knock‐down significantly decreased the expression of stathmin, while overexpression of PHAP1 increased it. Also, the upstream negative regulator, p27, expression levels increased upon PHAP1 knock‐down and decreased when PHAP1 was overexpressed. As a result, the phosphorylated Akt (S473), an upstream regulator of p27, expression levels decreased upon silencing of PHAP1, but elevated after PHAP1 overexpression. Importantly, we demonstrate the p27 down‐regulation, stathmin up‐regulation and cell proliferation acceleration induced by PHAP1 overexpression were dependent on Akt activation. In conclusion, the above results suggest that PHAP1 expression is elevated in glioma patients, which may accelerate the proliferation of glioma cells by regulating the Akt/p27/stathmin pathway.

## 1 INTRODUCTION

Glioma is the most common form of primary tumour that originates from the central nervous system.^1^ In line with the classification criteria of the World Health Organization (WHO), glioma is classified into lower grades (Grade I and II) and higher grades (Grade III and IV).^2^ In spite of significant progression in surgery and adjunctive therapy, the prognosis of glioma patients remains poor. The reported median survival time for patients diagnosed with glioma is about 15 months where less than 3 percent have a 5‐year survival rate.^3^ Therefore, investigating the mechanisms underlying gliomagenesis is critical to identify new biomarkers for earlier diagnostics, as well as develop novel targeted therapies to combat this highly malignant disease.

PHAP1 is a nuclear phosphoprotein that plays a variety of cellular roles in cell differentiation, gene transcription, cell apoptosis and cell cycle transition.^4^, ^5^, ^6^ Recent studies have shown PHAP1 plays important roles in human cancers. Most of the studies show that PHAP1 functions as a tumour suppressor in human breast cancer, pancreatic cancer and non‐small‐cell lung cancer.^7^, ^8^, ^9^ However, other reports demonstrate increased PHAP1 expression is linked to hepatocellular carcinoma, colorectal cancer, prostate cancers and oral squamous cell carcinoma,^10^, ^11^, ^12^, ^13^, ^14^ suggesting PHAP1 can also promote cancers. To date, the expression and function of PHAP1 in human glioma remain unknown.

Previous studies have demonstrated PHAP1 is involved in (1) the stabilization of mRNAs with AU‐rich elements (AREs) by interacting with the RNA‐binding protein HuR (ELAVL1);^5^ (2) the acetylation of histones by regulating the inhibitor of acetyl transferase complex (INHAT);^15^ and (3) the regulation of cell cycle by interacting with the phosphorylated Rb protein.^4^ Still, the function of PHAP1 needs to be further evaluated. Preliminary experiments by our group compared the protein expression between PHAP1 down‐regulated and control glioma cells by an iTRAQ proteomic analysis. The analysis demonstrated oncoprotein 18 (also called stathmin or STMN1), a marker of PI3K pathway activation, may be a potential downstream target of PHAP1.

Stathmin, as a mitotic regulator, plays important roles in maintaining the biological characteristics of the cells through controlling rapid microtubule remodelling.^16^, ^17^ Studies have shown that stathmin is expressed increasingly in several human malignancies, including leukaemia, mesothelioma, neuroblastoma and glioma.^18^, ^19^ Previous groups have reported that stathmin is negatively regulated by the cell cycle inhibitor p27.^20^ Moreover, Akt is a main regulator responsible for the modulation of p27.^21^ Based on the above findings, we hypothesized PHAP1 may regulate stathmin through the Akt/p27 pathway.

In this study, we investigate the role of PHAP1 in human glioma cells by assessing the expression of PHAP1 protein in glioma tissues and cell lines using Western blotting and immunohistochemistry analysis. Additionally, we determine PHAP1 plays a significant role in glioma cell proliferation. Finally, we explore the effects of PHAP1 on Akt/p27 regulated stathmin signalling.

## 2 MATERIALS AND METHODS

### 2.1 Antibodies

PHAP1 and stathmin antibodies were bought from Abcam (Cambridge, UK). Antibodies against Akt, p‐Akt (S473), p27 and β‐actin were purchased from Cell Signaling Technology (Danvers, MA, USA).

### 2.2 Tissue samples

Thirty specimens of human glioma tissues (Surgical resection) and twelve specimens of non‐tumour brain tissues (Internal decompression in cerebral trauma) were collected at the Affiliated Hospital of Xuzhou Medical University (Xuzhou, China). All glioma specimens have been confirmed by the pathological diagnosis and were classified in line with the criteria of WHO. Written informed consent was acquired from each patient, and the study was permitted by the Research Ethics Committee of Xuzhou Medical University.

### 2.3 Cell culture

Glioma cell lines C6, U251, U118, A172, U87 and human embryonic kidney cell line 293T were bought from Shanghai Cell Bank, Type Culture Collection Committee, Chinese Academy of Sciences. These cells were all supplemented with Dulbecco's modified Eagle's medium (DMEM) (Invitrogen, Carlsbad, CA, USA) containing 10% foetal bovine serum (TransGen, Beijing, China) and cultured in a humidified incubator with 5% CO_2_ at 37°C.

### 2.4 Constructs and production of the lentivirus

To knock‐down PHAP1, three sets of shRNA duplexes were designed and synthesized as the followings:

shPHAP1‐F1:

5′‐GATCGGACGCCCTCTGATGTGAATTCAAGAGATTCACATCAGAGGGCGTCCTTTTTTG‐3′

shPHAP1‐R1:

5′‐AATTCAAAAAAGGACGCCCTCTGATGTGAATCTCTTGAATTACATCAGAGGGCGTCC‐3′

shPHAP1‐F2:

5′‐GATCGCAAGACTCAGTGGTGTATTTCAAGAGAATACACCACTGAGTCTTGCTTTTTTG‐3′

shPHAP1‐R2:

5′‐AATTCAAAAAAGCAAGACTCAGTGGTGTATTCTCTTGAAATACACCACTGAGTCTTGC‐3′

shPHAP1‐F3:

5′‐GATCGACTCTGATGTTACTCTTGTTCAAGAGACAAGAGTAACATCAGAGTCTTTTTTG‐3′

shPHAP1‐R3:

5′‐AATTCAAAAAAGACTCTGATGTTACTCTTGTCTCTTGAACAAGAGTAACATCAGAGTC‐3′

Control‐F:

5′‐GATCTTCTCCGAACGTGTCACGTTTCAAGAGAACGTGACACGTTCGGAGAATTTTTTG‐3′

Control‐R

5′‐AATTCAAAAAATTCTCCGAACGTGTCACGTTCTCTTGAAACGTGACACGTTCGGAGAA‐3′

The PHAP1 shRNAs and control shRNA oligomers were annealed and then cloned into the pLV‐shRNA plasmid using the *BamH* I and *EcoR* I cloning sites. To overexpress PHAP1 in glioma cells, the PHAP1 cDNA was cloned into the pWPXLd‐puro plasmid by using *Bam*H I and *Mlu* I enzyme sites. Cell transfection was carried out by PolyJet (SignaGen, Gaithersburg, MD, USA) according to the manufacturer's instructions. The lentiviruses were produced by cotransfecting the core plasmid and the packaging plasmids in 293T cells.

### 2.5 Development of the stable cell lines

The stable cell lines were developed as we previously described.^22^, ^23^, ^24^ For stably knocking down or overexpressing PHAP1, the U251 and U87 cells were infected with the control, shPHAP1#3, GFP or GFP‐PHAP1 lentiviruses, respectively. Forty‐eight hours after infection, the cells were continuously provided with the medium supplemented with 2.5 μg/mL puromycin (Sigma, St. Louis, MO, USA). The survived cells were developed into stable cell lines that express control shRNA, shPHAP1 #3, GFP or GFP‐PHAP1.

### 2.6 Quantitative iTRAQ‐based proteomic analysis

Quantitative iTRAQ‐based proteomic analysis was performed by CapitalBio Technology Co. Ltd (Beijing, China). Total protein was extracted from U251‐Control, U251‐shPHAP1#3, U87‐Control and U87‐shPHAP1#3 cells. 100 μg of each protein was denatured in 8 mol/L urea in 50 mmol/L NH_4_HCO_3_ pH 7.4 and alkylated with 10 mmol/L iodoacetamide for 1 hour at 37°C. Then each sample was diluted 10‐fold with 25 mmol/L NH_4_HCO_3_ and digested with trypsin at a ratio of 1:100 (trypsin/substrate) for 6 hours at 37°C. A 25 μg aliquot of digested peptides for each sample was subjected to eight‐plex iTRAQ labelling according to the manufacturer's instructions. Peptides from each iTRAQ experiment were subjected to capillary liquid chromatography‐tandem mass spectrometry (LC‐MS/MS) using a Q Exactive Hybrid Quadrupole‐Orbitrap Mass Spectrometer (Thermo Fisher Scientific, CA, USA). The quantitative analysis was conducted by calculating the ratios between experimental group and control group. To make the data more credible, the iTRAQ experiment was repeated at three times. The changes were considered significant if the increased or decreased fold change >1.5 and the *P*‐value <.05. The original mass spectrum data were searched by database using Mascot 2.2 and Proteome Discoverer 1.4 (Thermo Fisher Scientific, CA, USA).

### 2.7 EdU assay

The cells stably knocking down or overexpressing PHAP1 were cultivated in 96‐well plates at 4 × 10^3^ cells/well. Twenty hours after culture, the cells were applied to 50 μmol/L of 5‐ethynyl‐20‐deoxyuridine (EdU; Ribobio, Guangzhou, China) and incubated for 2 hours at 37°C. The cells were washed with PBS and fixed with 4% paraformaldehyde for 20 minutes, and then permeabilized with 0.5% Triton X‐100 for another 20 minutes. Afterwards, the cells were washed five times with PBS and incubated with 100 μL of 1 × Apollo® reaction cocktail for 30 minutes at room temperature. Finally, the nuclei of the cells were dyed with 100 μL of Hoechst 33342 (5 μg/mL) for 20 minutes and visualized with a fluorescent microscopy (IX71; Olympus, Tokyo, Japan).

### 2.8 Colony formation assay

The plate colony formation assay was carried as we previously described.^22^, ^23^, ^24^ 5 mL of cell suspension containing 200 cells was inoculated into a diameter 60 mm dish for continuous culture until the visible clones appeared. After washing with PBS, the cells were fixed with 100% methanol. Afterwards, the cells were incubated with 0.5% crystal violet to subject for colony staining. Finally, the dishes were dried naturally and photographed with a camera for colony counting.

### 2.9 CCK‐8 assay

Two thousand cells in 100 μL of medium were cultured in several 96‐well plates. At the designated time‐point, 10 μL of CCK‐8 reagent was applied into the medium. After reaction for 4 hours at 37°C, the absorbance at 450 nm was determined by a SynergyMx Multi‐Mode Microplate Reader (Biotek, Winooski, VT). The cell viability was calculated according to the absorbance.

### 2.10 Western blotting

At the designated time‐point, the cells were harvested and subjected for total protein extraction. To analyse the protein expression by Western blotting, equal amounts of proteins were isolated on a 12% SDS‐PAGE and then transferred to 0.45 μm pore size PVDF membrane (Millipore, Billerica, MA, USA). After blocking with 3% bovine serum albumin (BSA), the membrane was incubated with the primary antibodies (PHAP1, Stathmin, Akt, Akt (S473), p27 and β‐actin) at 4°C overnight. On the following day, the membranes were probed with a horseradish peroxidase (HRP)‐labelled goat anti‐rabbit/mouse IgG. Bound antibodies were detected by the ECL Plus Western Blotting Substrate (Thermo Fisher, Waltham, MA, USA) and then visualized with a ChemiDoc™ Imaging System (Biorad, Hercules, CA, USA). Band density was quantified by Image J Software (Wayne Rasband, National Institutes of Health, MD). The relative amount of each protein was determined by normalizing the densitometry value of interest to that of the loading control.

### 2.11 Statistical analysis

The results shown were representative of experiments that were repeated at least three times. All quantitative data were presented as mean ± SEM. Statistical analysis was performed with the SPSS Version 13.0 (SPSS Inc, Chicago, IL). Differences in multiple groups were compared by a one‐way analysis of variance (ANOVA) followed by post hoc test. Differences between two groups were determined by Student's *t* test. *P* values <.05 were considered statistically significant (*P <* *.05).

## 3 RESULTS

### 3.1 PHAP1 protein is up‐regulated in human glioma patients and glioma cells

**Figure 1:**
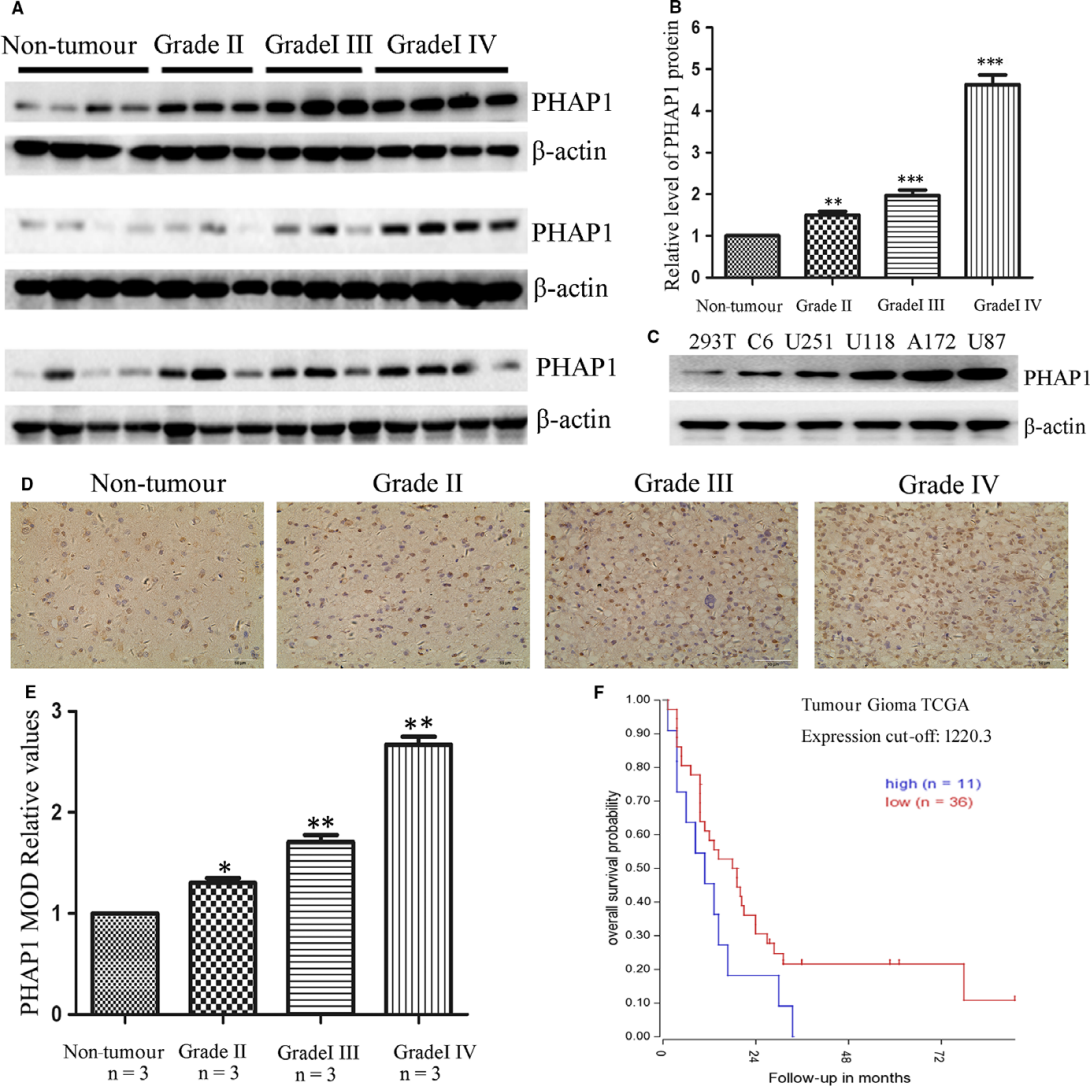
Expression of PHAP1 in human glioma patients and glioma cell lines. A, Total proteins isolated from non‐neoplastic brain tissues and glioma tissues were analysed by Western blotting for assessment of PHAPl. B, Statistical chart showed the expression level of PHAP1 in non‐tumourous brain tissue and the different grades of glioma tissues. The ratios indicate the levels of PHAP1 to β‐actin levels with respect to each sample. C, Expression of PHAP1 in non‐tumourous cell line (293T) and glioma cell lines (C6, U251, U118, A172, U87). D, Representative images and E, quantification of the immunohistochemical analysis showed PHAP1 was up‐regulated in human glioma patients. F, Kaplan‐Meier analysis with TCGA database showed higher level of PHAP1 was associated with poorer survival outcome in glioma patients. Scale bars: 50 μm. ∗, *P* < .05; ∗∗, *P* < .01; ∗∗∗, *P* < .001

To study the role of PHAP1 in the development of human gliomas, the total protein was isolated from 30 cases of human glioma tissue (9 cases of Grade II, 9 cases of Grade III, 12 cases of Grade IV) and 12 cases of non‐tumour brain tissue samples for Western blotting analysis. As shown in Figure 1A,B, the protein level of PHAP1 in glioma tissue was significantly increased compared with the non‐tumour brain tissue, especially in high‐grade glioma patients (grade III‐IV). In addition, we evaluated the PHAP1 expression level in non‐tumour cell line (293T) and several glioma cell lines (C6, U251, U118, A172 and U87). Our findings revealed the expression level of PHAP1 was elevated in glioma cell lines compared to non‐tumour cell lines which may correspond with glioma grade (Figure 1C). We also confirmed the protein expression of PHAP1 by immunohistochemical analysis, which was consistent with the Western blotting results (Figure 1D,E). Finally, we analysed the correlation between PHAP1 and patient survival utilizing the TCGA database. Glioma patients with elevated levels of PHAP1 were associated with poor prognosis. These data indicate that PHAP1 protein expression is highly expressed in human gliomas, providing initial evidence that PHAP1 may play an important role in the development and progression of human gliomas.

### 3.2 The effects of PHAP1 on glioma cell proliferation

**Figure 2:**
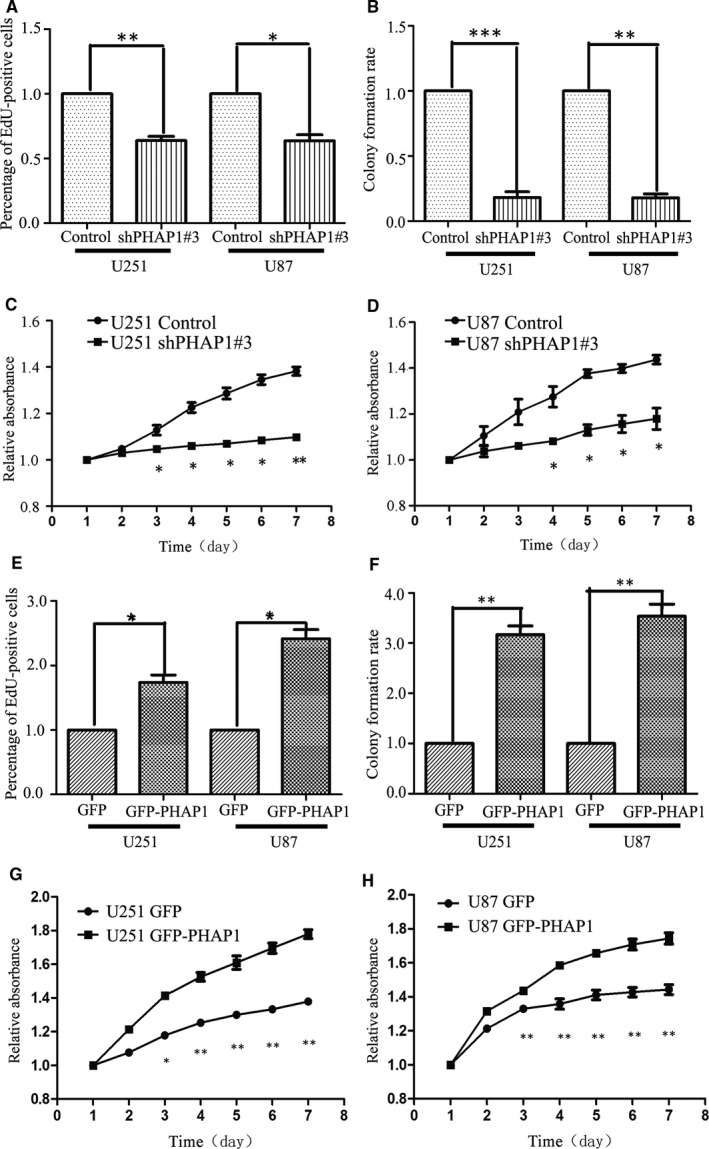
The effects of PHAP1 on glioma cell proliferation. A, EdU incorporation assay, colony formation assay B, and CCK‐8 assay (C and D) showed down‐regulation of PHAP1‐inhibited glioma cell proliferation in U251 and U87 cells. E, EdU incorporation assay, colony formation assay F, and CCK‐8 assay (G and H) showed overexpression of PHAP1‐promoted glioma cell proliferation in U251 and U87 cells

To explore the roles of PHAP1 in glioma cell proliferation, we used specific shRNAs to down‐regulate PHAP1 expression. To knock‐down PHAP1, three shRNA targets (shPHAP1#1, shPHAP1#2 and shPHAP1#3) were examined for their efficacy in suppressing PHAP1 expression, and a non‐targeting shRNA served as a negative control. ShPHAP1#3 was the ideal candidate for silencing PHAP1 expression (Figure S1A,B). To develop stable cell lines with PHAP1 knock‐down, shPHAP1#3 was used for lentivirus production and infection of the U251 and U87 cells. The stable cell lines were validated by GFP images (Figure S1C,D) and Western blotting (Figure S1E,F). Next, we examined whether the cell proliferation was affected by knocking down PHAP1. The EdU incorporation assay showed that EdU‐positive cells in the PHAP1 down‐regulated group decreased in U251 and U87 cells by 38.7% and 36.3%, respectively, compared with the control group (Figure S1G,H & Figure 2A). The capability of colony formation was also significantly inhibited by knocking down of PHAP1 (Figure S1I,J & Figure 2B). In addition, the CCK‐8 assay showed the cell viability was reduced upon knocking down PHAP1 compared to the control group (Figures 2C and D).

Additionally, we overexpressed GFP‐PHAP1 in U251 and U87 cells to further determine the function of PHAP1 in the proliferation of glioma cells. The expression efficiency of PHAP1 was confirmed by GFP images (Figure S2A,B) and Western blotting (Figure S2C,D). We then validated whether overexpression of PHAP1 could promote the cell proliferation. EdU incorporation assay showed that the number of proliferating cells of the GFP‐PHAP1 group was increased in U251 and U87 cells by 34% and 62%, respectively, compared with GFP group (Figure S2E,F & Figure 2E). The capability of colony formation was also significantly enhanced by overexpression of PHAP1 (Figure S2G,H & Figure 2F). In addition, CCK‐8 assay indicated that overexpression of PHAP1 significantly accelerated the proliferation of U251 and U87 cells compared to the control groups (Figure 2G,H). These data suggest that PHAP1 is important for the proliferation of human glioma cells, and down‐regulation of PHAP1 suppresses the proliferation of glioma cells, while overexpression of PHAP1 promotes it.

### 3.3 Identification of proteins regulated by PHAP1 via quantitative proteomic analysis

**Figure 3:**
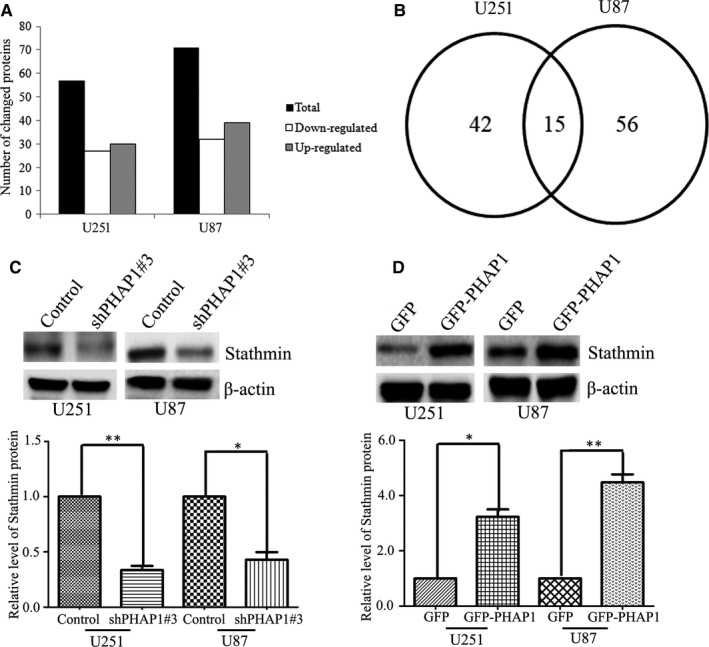
Identification of proteins regulated by PHAP1 via proteomic analysis. A, The bar chart showed the numbers of proteins that are significantly regulated by PHAP1. B, Venn diagram showed the number of overlapped proteins that significantly regulated by PHAP1. C, Representative blots and D, quantification showed the protein level of stathmin in PHAP1 silenced or overexpressed U251 and U87 cells. *P < .05, **P < .01

The above results have indicated that PHAP1 is important for the proliferation of glioma cells. However, the underlined mechanisms are not yet elucidated. Thus, we performed an iTRAQ proteomic analysis to compare the protein expression between control and shPHAP1#3 in both U251 and U87 cells. A total of 5400 peptides were detected in three independent biological replicates among four groups. Then, we analysed the identified peptides. As a result, 57 and 71 changed proteins were identified in U251 and U87 cells, respectively (Figure 3A). In U251 cells, there were 27 down‐regulated proteins and 30 up‐regulated proteins, however, in U87 cells, there were 32 down‐regulated proteins and 39 up‐regulated proteins (Figure 3A). Among them, 15 proteins overlapped in U251 and U87 cells (Figure 3B). Among the overlapped proteins, stathmin was one of the differentially expressed PHAP1 targets implicated in cell proliferation. Moreover, stathmin has been demonstrated to promote cancer cell proliferation in various cancers.^25^, ^26^, ^27^, ^28^, ^29^ Thus, we hypothesized that stathmin may be involved in PHAP1‐regulated glioma cell proliferation. We validated the iTRAQ results by Western blotting analysis which demonstrated that silencing of PHAP1 significantly decreased the expression of stathmin, while overexpression of PHAP1 increased expression (Figure 3C,D).

### 3.4 PHAP1 promotes glioma cell proliferation by regulating Akt/p27/stathmin pathway

**Figure 4:**
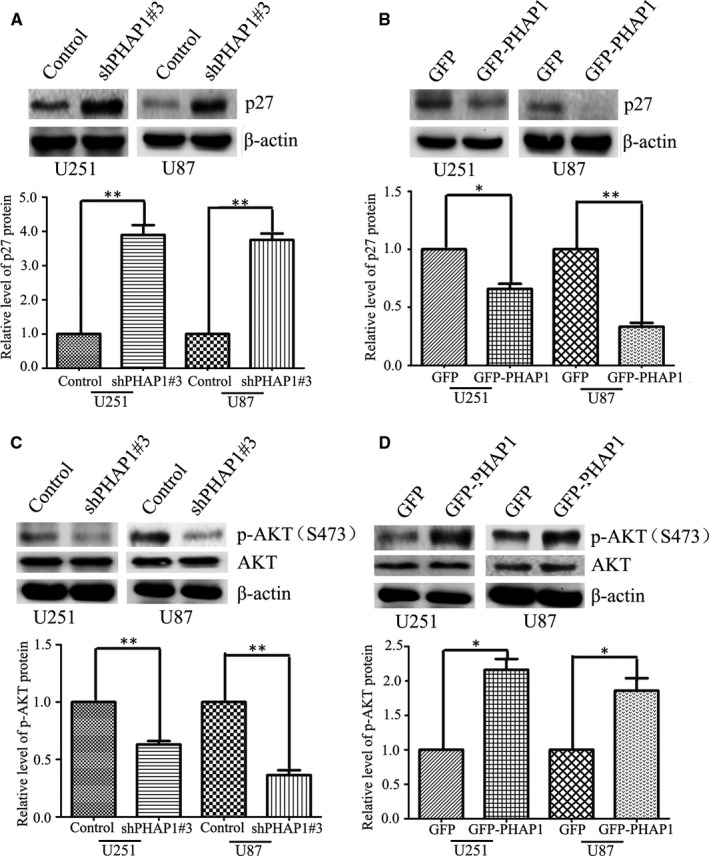
PHAP1 regulates stathmin by Akt/p27 pathway. A, Representative bolts and B, quantification to show the protein level of p27 in PHAP1‐silenced or PHAP1‐overexpressed U251 and U87 cells. C, Representative blots and D, quantification to show the level of total Akt and phosphorylated Akt in PHAP1‐silenced or PHAP1‐overexpressed U251 and U87 cells. *P < .05, **P < .01

**Figure 5:**
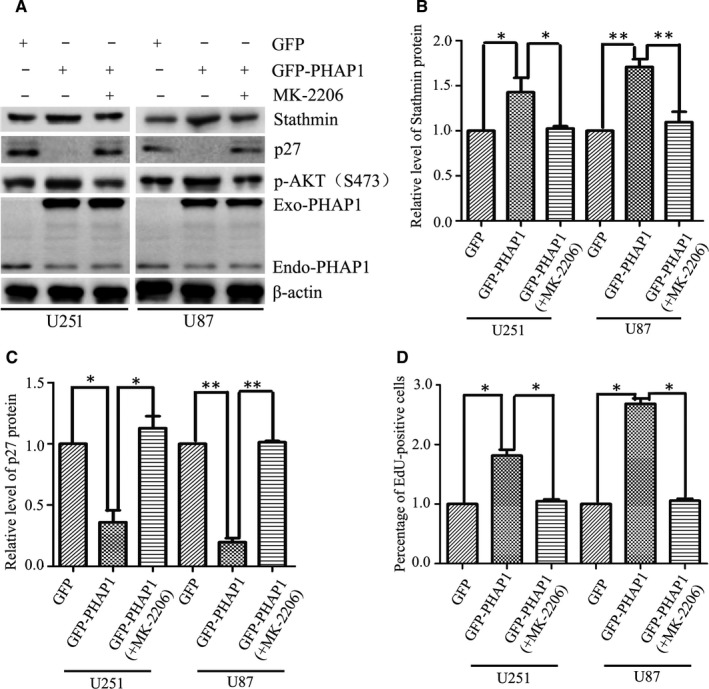
Akt inhibitor significantly blocks PHAP1 overexpression‐induced p27 down‐regulation, stathmin up‐regulation and cell proliferation. A, Representative blots and (B and C) quantification showed that Akt inhibitor MK‐2206 effectively attenuated PHAP1 overexpression‐induced p27 down‐regulation and stathmin up‐regulation in U251 and U87 cells. D, EdU assay showed Akt inhibitor significantly blocked PHAP1 overexpression‐induced glioma cell proliferation. *P < .05, **P < .01

Previous reports show stathmin is negatively controlled by the cell cycle inhibitor p27.^20^ Therefore, we assessed the expression of p27 in the PHAP1‐modulated cells. As expected, the upstream negative regulator p27 was increased in PHAP1 down‐regulated U251 and U87 cells, whereas decreased in PHAP1 overexpressed U251 and U87 cells (Figure 4A,B). Consistently, phosphorylated Akt (S473), the upstream regulator of p27, was decreased upon knocking down PHAP1, but enhanced when PHAP1 was overexpressed (Figure 4C,D). Most importantly, to confirm PHAP1 and Akt/p27/stathmin are in the same pathway, the Akt inhibitor MK‐2206 was used to perform the rescue experiment. Overexpressing of PHAP1 in U251 and U87 cells significantly up‐regulated levels of p‐Akt (S473) thereby down‐regulating the p27 and increasing the stathmin. However, this regulation could be significantly blocked by the Akt inhibitor (Figure 5A‐C). These results indicate that stathmin up‐regulation induced by PHAP1 overexpression is largely attributed to the activation of Akt. In addition, a rescue experiment was also conducted to confirm the above results by an EdU assay in the presence of the Akt inhibitor. It was shown that glioma cell proliferation induced by PHAP1 overexpression was also dependent on the Akt activation (Figure S3 & Figure 5D). Taken together, these findings indicate that PHAP1 could promote the proliferation of glioma cells by regulating the Akt/p27/stathmin pathway.

## 4 DISCUSSION

PHAP1 is a multi‐functional phosphoprotein that is involved in multiple cellular processes, including cell differentiation, gene transcription, apoptosis and cell cycle transition.^4^, ^30^, ^31^ It has been long considered that PHAP1 functions as a tumour inhibitor in several human cancers, such as pancreatic cancer, breast cancer and non‐small‐cell lung cancer.^7^, ^8^, ^9^ However, new findings reveal PHAP1 may also act as an oncoprotein in some human cancers, including hepatocellular carcinoma, colorectal cancer, prostate cancers and oral squamous cell carcinoma.^10^, ^11^, ^12^, ^13^, ^14^ Still, limited information about PHAP1 expression and its clinical relevance in glioma patients remains unclear. In our present study, we evaluated the protein expression of PHAP1 in glioma tissue and normal brain tissues by Western blotting and immunohistochemistry staining. We discovered that PHAP1 protein was expressed at high levels in glioma patients, especially in those with high‐grade malignancy, compared to tissues derived from normal brain. Interestingly, the expression level of PHAP1 was also higher in glioma cell lines than in non‐tumour cell lines. Additionally, even more abundant PHAP1 levels were observed in the U87 cell line which represents high‐grade malignancy. More importantly, our findings revealed higher levels of PHAP1 was associated with poor survival in glioma patients. Further functional experiments demonstrated that knock‐down of PHAP1 inhibited cell proliferation of glioma cells, whereas overexpression of PHAP1 promoted it. These results suggest that PHAP1 functions as an oncoprotein in the development and progression of glioma, which may serve as a potential biomarker for glioma.

Depression of specific tumour suppressor genes or activation of specific oncogenes is a key event for the development and malignant progression of glioblastoma.^32^, ^33^, ^34^ Particularly, the molecules modulating the expression of PI3K/Akt pathway are important for the survival of cancer cells.^35^ Akt has been regarded as a very valuable therapeutic target in glioma due to the alteration of multiple signalling pathways in glioma that converge to Akt, as well as, the aggressive characteristics of glioma that regulate genes involved in cell proliferation, apoptosis, migration, invasion and neoangiogenesis.^36^, ^37^ The excessive activation of Akt signalling in glioma is therefore facilitated by abnormal signals from upstream regulators.^23^, ^38^, ^39^ This usually includes gain‐of‐function mutations or up‐regulation of the upstream activators, or loss‐of‐function mutation or down‐regulation of the upstream inhibitors. PHAP1 has been demonstrated to regulate HuR, inhibitor of acetyl transferase complex (INHAT) and Rb to function as a tumour suppressor or an oncoprotein.^4^, ^5^, ^15^ In this study, we found that PHAP1 could promote glioma cell proliferation by modulating Akt pathway. This demonstrates PHAP1 may be involved in the development and progression of glioma by contributing to the Akt pathway. Increased expression of PHAP1 may be a critical factor leading to the activation of the Akt pathway in human glioma. Taken together, we can conclude that higher expression of PHAP1 in glioma patients may cause the abnormal activation of Akt‐dependent stathmin signalling, which facilitates glioma cell proliferation. Therefore, aberrant expression of PHAP1 may be an important event in the development and malignant progression of glioma.

Stathmin regulates dynamic instability, the growth and shrinkage of microtubules by modulating microtubule plus end catastrophes and sequestering alpha‐beta tubulin dimers.^40^ Consequently, stathmin has direct effects on cellular processes including cell growth and motility by influencing the association of microtubules with the actin cytoskeleton.^41^ Consistent with this, stathmin has been shown to play important roles in glioma cell growth and motility.^19^, ^42^, ^43^ In our study, we found that the stathmin was significantly up‐regulated upon PHAP1‐induced activation of Akt pathway. Furthermore, the up‐regulation of stathmin expression and acceleration of glioma cell proliferation induced by PHAP1 overexpression could be almostly blocked by Akt inhibitor. These results indicate that the up‐regulation of stathmin expression and glioma cell proliferation acceleration induced by PHAP1 overexpression is mainly mediated by the Akt pathway.

In conclusion, we demonstrate how PHAP1 is linked to malignant proliferation of human glioma through the Akt/p27/stathmin pathway. Our work elucidates new findings that warrant further exploration of the PHAP1‐mediated signalling pathway, as well as, evaluation of the prognostic potential of PHAP1 expression in glioma patients. However, deeper investigations are still needed to elaborate how PHAP1 to regulate the activation of Akt in human glioma in the future study.

## CONFLICT OF INTEREST

The authors declare that they have no conflict of interest.

## References

[1] Ostrom QT , Gittleman H , Stetson L , Virk SM , Barnholtz‐Sloan JS . Epidemiology of gliomas. Cancer Treat Res. 2015;163:1‐14. 25468222 10.1007/978-3-319-12048-5_1

[2] Louis DN , Perry A , Reifenberger G , et al. The 2016 World Health Organization Classification of Tumors of the Central Nervous System: a summary. Acta Neuropathol. 2016;131:803‐820. 27157931 10.1007/s00401-016-1545-1

[3] Shahar T , Rozovski U , Hess KR , et al. Percentage of mesenchymal stem cells in high‐grade glioma tumor samples correlates with patient survival. Neuro Oncol. 2017;19:660‐668. 28453745 10.1093/neuonc/now239 PMC5464439

[4] Adegbola O , Pasternack GR . Phosphorylated retinoblastoma protein complexes with pp32 and inhibits pp32‐mediated apoptosis. J Biol Chem. 2005;280:15497‐15502. 15716273 10.1074/jbc.M411382200

[5] Williams TK , Costantino CL , Bildzukewicz NA , et al. pp32 (ANP32A) expression inhibits pancreatic cancer cell growth and induces gemcitabine resistance by disrupting HuR binding to mRNAs. PLoS One. 2010;5:e15455. 21152064 10.1371/journal.pone.0015455 PMC2994932

[6] Kadota S , Nagata K . pp32, an INHAT component, is a transcription machinery recruiter for maximal induction of IFN‐stimulated genes. J Cell Sci. 2011;124:892‐899. 21325029 10.1242/jcs.078253

[7] Bai J , Brody JR , Kadkol SS , Pasternack GR . Tumor suppression and potentiation by manipulation of pp32 expression. Oncogene. 2001;20:2153‐2160. 11360199 10.1038/sj.onc.1204294

[8] Schafer ZT , Parrish AB , Wright KM , et al. Enhanced sensitivity to cytochrome c‐induced apoptosis mediated by PHAPI in breast cancer cells. Cancer Res. 2006;66:2210‐2218. 16489023 10.1158/0008-5472.CAN-05-3923

[9] Hoffarth S , Zitzer A , Wiewrodt R , et al. pp32/PHAPI determines the apoptosis response of non‐small‐cell lung cancer. Cell Death Differ. 2008;15:161‐170. 17962813 10.1038/sj.cdd.4402256

[10] Li C , Ruan HQ , Liu YS , et al. Quantitative proteomics reveal up‐regulated protein expression of the SET complex associated with hepatocellular carcinoma. J Proteome Res. 2012;11:871‐885. 22082227 10.1021/pr2006999

[11] Shi H , Hood KA , Hayes MT , Stubbs RS . Proteomic analysis of advanced colorectal cancer by laser capture microdissection and two‐dimensional difference gel electrophoresis. J Proteomics. 2011;75:339‐351. 21843667 10.1016/j.jprot.2011.07.025

[12] Yan W , Bai Z , Wang J , Li X , Chi B , Chen X . ANP32A modulates cell growth by regulating p38 and Akt activity in colorectal cancer. Oncol Rep. 2017;38:1605‐1612. 28731192 10.3892/or.2017.5845

[13] Kadkol SS , Brody JR , Epstein JI , Kuhajda FP , Pasternack GR . Novel nuclear phosphoprotein pp32 is highly expressed in intermediate‐ and high‐grade prostate cancer. Prostate. 1998;34:231‐237. 9492852 10.1002/(sici)1097-0045(19980215)34:3<231::aid-pros11>3.0.co;2-f

[14] Velmurugan BK , Yeh KT , Lee CH , et al. Acidic leucine‐rich nuclear phosphoprotein‐32A (ANP32A) association with lymph node metastasis predicts poor survival in oral squamous cell carcinoma patients. Oncotarget. 2016;7:10879‐10890. 26918356 10.18632/oncotarget.7681 PMC4905446

[15] Higashino F , Aoyagi M , Takahashi A , et al. Adenovirus E4orf6 targets pp32/LANP to control the fate of ARE‐containing mRNAs by perturbing the CRM1‐dependent mechanism. J Cell Biol. 2005;170:15‐20. 15983058 10.1083/jcb.200405112 PMC2171388

[16] Lin X , Liu S , Luo X , et al. EBV‐encoded LMP1 regulates Op18/stathmin signaling pathway by cdc2 mediation in nasopharyngeal carcinoma cells. Int J Cancer. 2009;124:1020‐1027. 19048596 10.1002/ijc.23767

[17] Rubin CI , Atweh GF . The role of stathmin in the regulation of the cell cycle. J Cell Biochem. 2004;93:242‐250. 15368352 10.1002/jcb.20187

[18] Rana S , Maples PB , Senzer N , Nemunaitis J . Stathmin 1: a novel therapeutic target for anticancer activity. Expert Rev Anticancer Ther. 2008;8:1461‐1470. 18759697 10.1586/14737140.8.9.1461

[19] Dong B , Mu L , Qin X , et al. Stathmin expression in glioma‐derived microvascular endothelial cells: a novel therapeutic target. Oncol Rep. 2012;27:714‐718. 22038457 10.3892/or.2011.1525

[20] Fabris L , Berton S , Pellizzari I , et al. p27kip1 controls H‐Ras/MAPK activation and cell cycle entry via modulation of MT stability. Proc Natl Acad Sci USA. 2015;112:13916‐13921. 26512117 10.1073/pnas.1508514112 PMC4653222

[21] Shin I , Yakes FM , Rojo F , et al. PKB/Akt mediates cell‐cycle progression by phosphorylation of p27(Kip1) at threonine 157 and modulation of its cellular localization. Nat Med. 2002;8:1145‐1152. 12244301 10.1038/nm759

[22] Zou S , Zhu Y , Wang B , et al. The ubiquitin ligase COP1 promotes glioma cell proliferation by preferentially downregulating tumor suppressor p53. Mol Neurobiol. 2017;54:5008‐5016. 27534417 10.1007/s12035-016-0033-x

[23] Zhu Y , Zhang X , Wang L , et al. Loss of SH3GL2 promotes the migration and invasion behaviours of glioblastoma cells through activating the STAT3/MMP2 signalling. J Cell Mol Med. 2017;21:2685‐2694. 28470949 10.1111/jcmm.13184 PMC5661104

[24] Zhu Y , Qiu Z , Zhang X , et al. Jab1 promotes glioma cell proliferation by regulating Siah1/beta‐catenin pathway. J Neurooncol. 2017;131:31‐39. 27640199 10.1007/s11060-016-2279-6

[25] Reyes HD , Miecznikowski J , Gonzalez‐Bosquet J , et al. High stathmin expression is a marker for poor clinical outcome in endometrial cancer: an NRG oncology group/gynecologic oncology group study. Gynecol Oncol. 2017;146:247‐253. 28532857 10.1016/j.ygyno.2017.05.017 PMC5526627

[26] Ghosh R , Gu G , Tillman E , et al. Increased expression and differential phosphorylation of stathmin may promote prostate cancer progression. Prostate. 2007;67:1038‐1052. 17455228 10.1002/pros.20601

[27] Rong B , Nan Y , Liu H , Gao W . Increased stathmin correlates with advanced stage and poor survival of non‐small cell lung cancer. Cancer Biomark. 2017;19:35‐43. 28282798 10.3233/CBM-160239 PMC13020710

[28] Nie W , Xu MD , Gan L , Huang H , Xiu Q , Li B . Overexpression of stathmin 1 is a poor prognostic biomarker in non‐small cell lung cancer. Lab Invest. 2015;95:56‐64. 25384122 10.1038/labinvest.2014.124

[29] Belletti B , Baldassarre G . Stathmin: a protein with many tasks. New biomarker and potential target in cancer. Expert Opin Ther Targets 2011;15:1249‐1266. 21978024 10.1517/14728222.2011.620951

[30] Theodosiou A , Ashworth A . MAP kinase phosphatases. Genome Biol. 2002;3:REVIEWS300. 10.1186/gb-2002-3-7-reviews3009 PMC139386 12184814

[31] Chakravarti D , Hong R . SET‐ting the stage for life and death. Cell. 2003;112:589‐591. 12628178 10.1016/s0092-8674(03)00151-x

[32] Tung JN , Ko CP , Yang SF , et al. Inhibition of pentraxin 3 in glioma cells impairs proliferation and invasion in vitro and in vivo. J Neurooncol. 2016;129:201‐209. 27278519 10.1007/s11060-016-2168-z

[33] Yue C , Niu M , Shan QQ , et al. High expression of Bruton's tyrosine kinase (BTK) is required for EGFR‐induced NF‐kappaB activation and predicts poor prognosis in human glioma. J Exp Clin Cancer Res. 2017;36:132. 28946903 10.1186/s13046-017-0600-7 PMC5613332

[34] Renner G , Noulet F , Mercier MC , et al. Expression/activation of alpha5beta1 integrin is linked to the beta‐catenin signaling pathway to drive migration in glioma cells. Oncotarget. 2016;7:62194‐62207. 27613837 10.18632/oncotarget.11552 PMC5308720

[35] Holand K , Salm F , Arcaro A . The phosphoinositide 3‐kinase signaling pathway as a therapeutic target in grade IV brain tumors. Curr Cancer Drug Targets. 2011;11:894‐918. 21861842 10.2174/156800911797264743

[36] Chautard E , Loubeau G , Tchirkov A , et al. Akt signaling pathway: a target for radiosensitizing human malignant glioma. Neuro Oncol. 2010;12:434‐443. 20406894 10.1093/neuonc/nop059 PMC2940626

[37] Nan Y , Guo L , Song Y , et al. Combinatorial therapy with adenoviral‐mediated PTEN and a PI3K inhibitor suppresses malignant glioma cell growth in vitro and in vivo by regulating the PI3K/AKT signaling pathway. J Cancer Res Clin Oncol. 2017;143:1477‐1487. 28401302 10.1007/s00432-017-2415-5 PMC11819009

[38] Jia P , Li F , Gu W , Zhang W , Cai Y . Gab3 overexpression in human glioma mediates Akt activation and tumor cell proliferation. PLoS One. 2017;12:e0173473. 28291820 10.1371/journal.pone.0173473 PMC5349442

[39] Gao S , Wang J , Zhang T , et al. Low expression of CAPON in glioma contributes to cell proliferation via the Akt signaling pathway. Int J Mol Sci. 2016;17:pii: E1859. 10.3390/ijms17111859 PMC5133859 27869735

[40] Cassimeris L . The oncoprotein 18/stathmin family of microtubule destabilizers. Curr Opin Cell Biol. 2002;14:18‐24. 11792540 10.1016/s0955-0674(01)00289-7

[41] Rodriguez OC , Schaefer AW , Mandato CA , Forscher P , Bement WM , Waterman‐Storer CM . Conserved microtubule‐actin interactions in cell movement and morphogenesis. Nat Cell Biol. 2003;5:599‐609. 12833063 10.1038/ncb0703-599

[42] Song Y , Mu L , Han X , et al. MicroRNA‐9 inhibits vasculogenic mimicry of glioma cell lines by suppressing Stathmin expression. J Neurooncol. 2013;115:381‐390. 24043603 10.1007/s11060-013-1245-9

[43] Liang XJ , Choi Y , Sackett DL , Park JK . Nitrosoureas inhibit the stathmin‐mediated migration and invasion of malignant glioma cells. Cancer Res. 2008;68:5267‐5272. 18593927 10.1158/0008-5472.CAN-07-6482 PMC2493613

