# Supplementary figures and images for: PHAP1 promotes glioma cell proliferation by regulating the Akt/p27/stathmin pathway
Source: J Cell Mol Med. 2018 Apr 18;22(7):3595–604. doi: 10.1111/jcmm.13639 (PMC6033192; doi:10.1111/jcmm.13639)

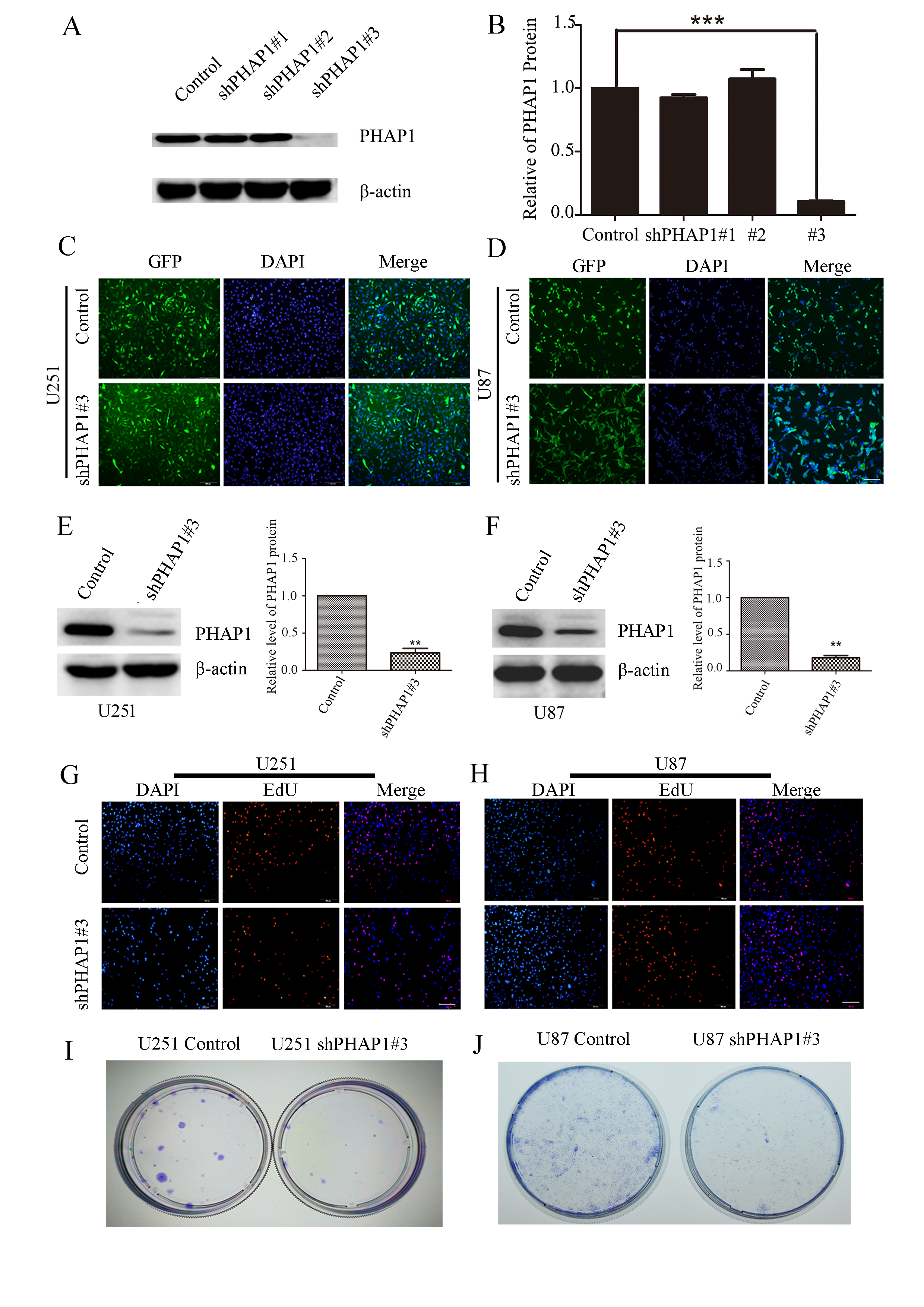

Supplement: Supplementary file 1 [file JCMM-22-3595-s001.tif]

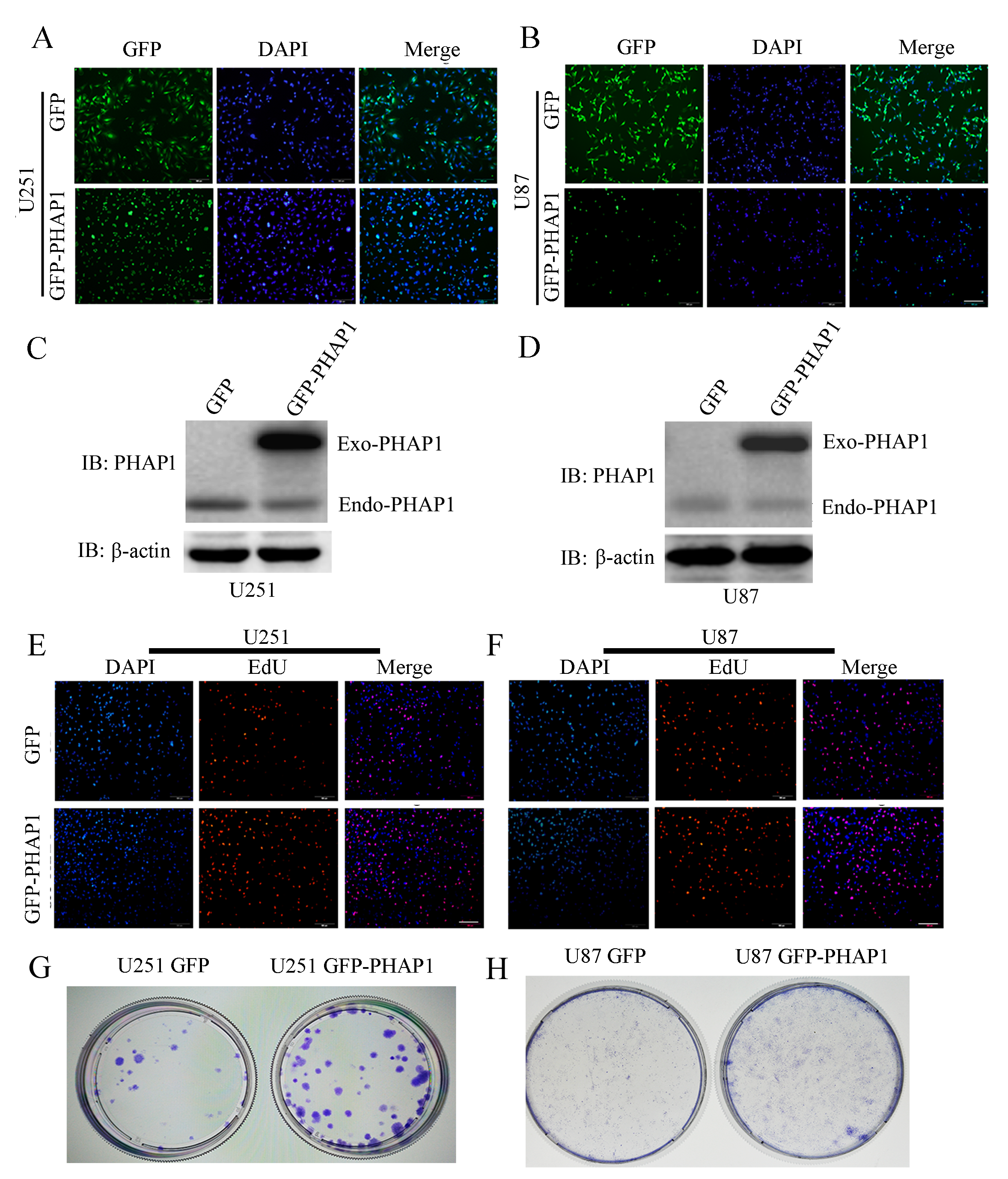

Supplement: Supplementary file 2 [file JCMM-22-3595-s002.tif]

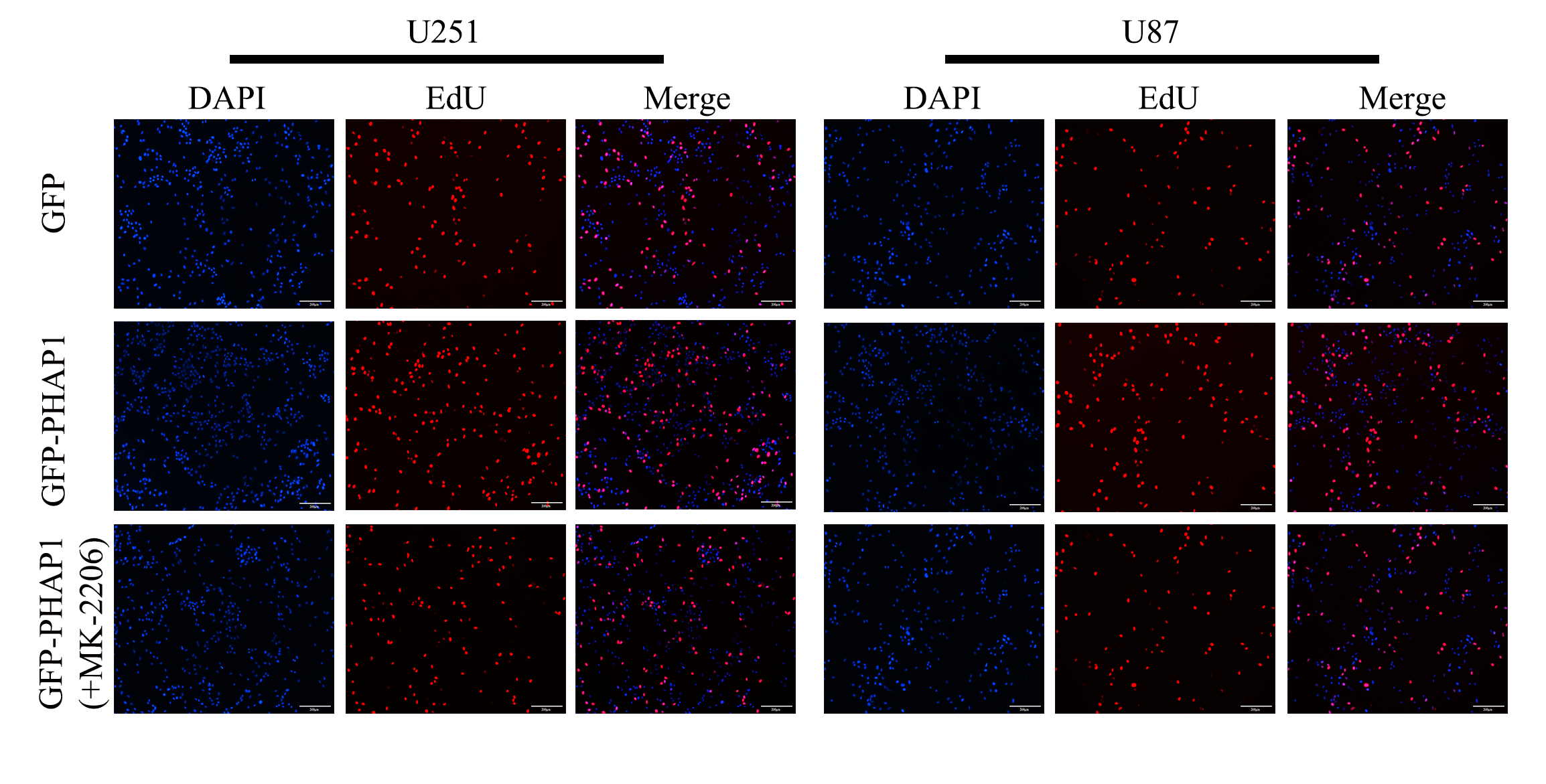

Supplement: Supplementary file 3 [file JCMM-22-3595-s003.tif]
